# Comparative Study of Different Polymeric Binders in Electrochemical CO Reduction

**DOI:** 10.1021/acs.energyfuels.4c04058

**Published:** 2024-11-05

**Authors:** Noémi V. Galbicsek, Attila Kormányos, Gergely Ferenc Samu, Mohd M. Ayyub, Tomaž Kotnik, Sebastijan Kovačič, Csaba Janáky, Balázs Endrődi

**Affiliations:** † Department of Physical Chemistry and Materials Science, University of Szeged, Rerrich Square 1, Szeged H-6720, Hungary; ‡ ELI-ALPS, ELI-HU Non-Profit Ltd., Wolfgang Sandner Street 3, 6728, Szeged H-6728, Hungary; § Department of Molecular and Analytical Chemistry, University of Szeged, Dóm Square 7-8, Szeged H-6721, Hungary; ∥ National Institute of Chemistry, Hajdrihova 19, Ljubljana SI-1001, Slovenia; ⊥ Faculty of Chemistry and Chemical Engineering, University of Maribor, Smetanova 17, Maribor SI-2000, Slovenia

## Abstract

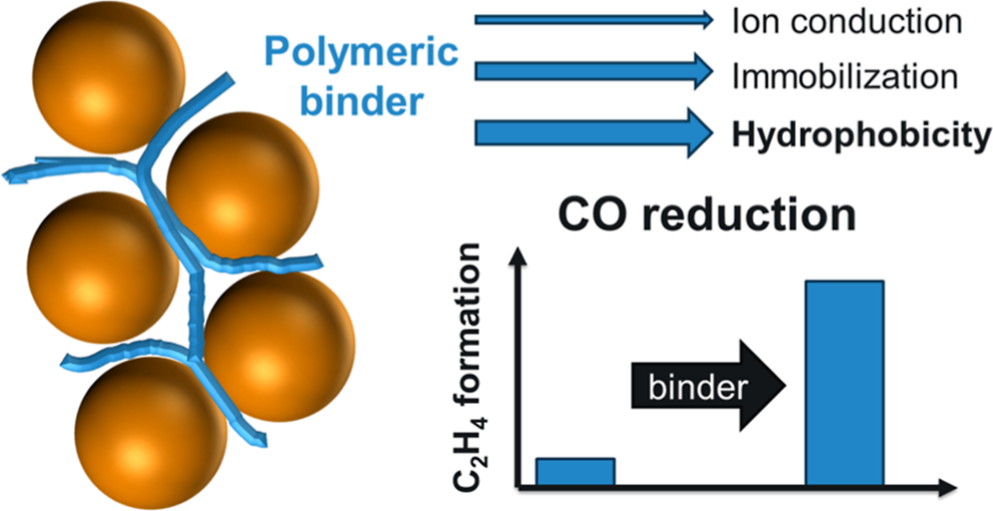

Electrochemical reduction of carbon monoxide offers a possible route to produce valuable chemicals (such as acetate, ethanol or ethylene) from CO_2_ in two consecutive electrochemical reactions. Such deeply reduced products are formed via the transfer of 4–6 electrons per CO molecule. Assuming similar-sized CO_2_ and CO electrolyzers, 2–3-times larger current densities are required in the latter case to match the molar fluxes. Such high reaction rates can be ensured by tailoring the structure of the gas diffusion electrodes. Here, the structure of the cathode catalyst layer was systematically varied using different polymeric binders to achieve high reaction rates. Simple linear polymers, bearing the same backbone but different functional groups were compared to highlight the role of different structural motifs. The comparison was also extended to simple linear, partially fluorinated polymers. Interestingly, in some cases similar results were obtained as with the current state-of-the-art binders. Using different surface-wetting characterization techniques, we show that the hydrophobicity of the catalyst layer—provided by the binder— is a prerequisite for high-rate CO electrolysis. The validity of this notion was demonstrated by performing CO electrolysis experiments at high current density (1 A cm^–2^) for several hours using PVDF as the catalyst binder.

## Introduction

Electrochemical synthesis has become increasingly important in recent years. This includes the synthesis of various high-value fine chemicals, but the production of bulk chemicals is also considered.^1,2^ Beyond the traditional chloride transformation chemistries,^3,4^ which still take a major share from the electricity consumption of electrosynthesis procedures, the volume of electrochemical hydrogen production also increases steeply.^5,6^ Based on the scientific interest in the field, a similarly rapid spreading of electrochemical CO_2_ reduction (CO_2_RR) technology is expected, once questions regarding stability, selectivity, and scale-up are properly addressed.^7−11^ CO_2_RR is appealing for many reasons, including reducing atmospheric CO_2_ emissions at point sources and forming valuable carbon-based raw materials (e.g., CO, C_2_H_4_, etc.), while storing renewable energy.^12^

The selective production of CO at high rates has been demonstrated using laboratory-scale devices, and therefore the scale-up^13^ of this technology is expected to be the flagship of industrial CO_2_RR. Several (semi)pilot scale systems are being developed simultaneously in various research institutes and companies.^14^ Carbon monoxide can be used in catalytic processes (e.g., methanol synthesis) and is therefore a valuable raw material. An alternative is its further electrochemical reduction (CORR), which aims to form even more valuable chemicals, such as ethylene. Noteworthy, electrochemical ethylene production could ideally be carried out directly from CO_2_, in one-step, but the selective and stable formation of multicarbon products has yet to be achieved in CO_2_RR. A two-step formation of these products via CO intermediate could be advantageous allowing the use of specifically optimized catalysts in the two reaction steps so that the cell could be operated with higher energy efficiency and/or at higher rate.^8^ Another advantage could be a reduction of reactant losses, an often-mentioned bottleneck in CO_2_RR, which is exacerbated when more deeply reduced products are formed.^15^ Although the importance of this aspect depends strongly on the energy cost for the separation of the anodic O_2_/CO_2_ mixture (and therefore it might be overestimated in some cases),^16,17^ the study of CORR still has its importance in the selective formation of different multicarbon products, at current densities that are notably higher compared to the case of CO_2_RR.^18^

Carbon monoxide is a gas of very low water solubility hence, its electrochemical reduction at high rates is only conceivable in certain solvents or under conditions where the diffusion length in the solution phase is minimized.^19^ Ideally, a large three-phase-boundary area should be ensured, at which the CO from the gas phase meets a sufficient amount of liquid water on the surface of the solid catalyst.^20^ A more realistic scenario is to minimize the solvation layer thickness at the catalyst layer, which ensures a short diffusion length (and reduced mass transport limitations). In continuous-flow electrolyzers, where CO gas is fed to the cathode, this can be influenced by many experimental parameters, including the gas flow rate, temperature, and pressure.^21−25^ The properties of the porous substrate—the gas diffusion layer—on which the catalyst layer is deposited to ensure a large accessible surface area, are even more important, also influencing the water management in the cell.^26^

In addition to these, the key to efficient CORR is the catalyst layer on which the actual CO reduction process takes place. Despite efforts to identify catalysts that are active (and selective) in CORR, only copper and copper-based systems appear to be suitable so far.^27,28^ Recently, the importance of the microstructure of the catalyst layer has also attracted increasing attention; by using suitable catalyst binders, the achievable product formation rates can be increased significantly.^29−34^ This idea is very much in line with the extensive literature on PEM fuel-cells (PEMFC). These studies on tuning the local catalyst microenvironment may be more relevant to CORR than the studies on CO_2_RR, as H_2_ and CO are almost equally insoluble gases, posing similar challenges. A notable difference that must be considered is the cocatalytic role of alkali metal cations in CORR.^35^ Their presence on the cathode catalyst surface must be ensured in sufficient concentration and without excessive amounts of water.

A variety of additives have already been tested in PEMFC electrodes, including different inert polymers, polyelectrolytes, functional molecules, or inorganic additives.^36−38^ In fact, these additives can serve multiple purposes, including the physical binding of the catalyst particles on the porous substrate, ensuring a porous catalyst layer structure, affecting the local chemical conditions, and participating in the ion conduction. In what follows, we will simply refer to these additives as “binders”.

One of the most important roles of these additives in electrolysis applications is to limit the amount of water on the catalyst surface and thus prevent the pores in the catalyst layer and the porous substrate from filling up (referred to as flooding), which would limit the electrochemically active surface area and thus the reaction rate. The binder can also participate in the electrochemical reactions by physically/chemically binding the reactants/intermediates, influencing the mass transport to the catalyst surface.^39,40^ However, this can only happen if the reactant is brought to the catalysts surface. Therefore, we attribute a similar requirement for the binder in CORR as in PEMFCs, namely that it must ensure a very short diffusion length for the reactant to reach the catalytically active centers. We argue that the investigation of tailor-designed macromolecules/polymers as binders must be preceded by studies on simplified systems.

Building on our previous results highlighting Capstone ST-110 as a potential binder for CORR,^41^ we have designed and investigated different polymeric binders in CORR that mimic different structural elements (backbone, functional group) of this complex, triblock copolymer. To further simplify the polymer structure, we also performed experiments with linear, fluorinated polymers and compared all these results with those measured using either Capstone ST-110 or Nafion (very often applied in CORR studies).

## Experimental Section

### Materials

**Figure 1:**
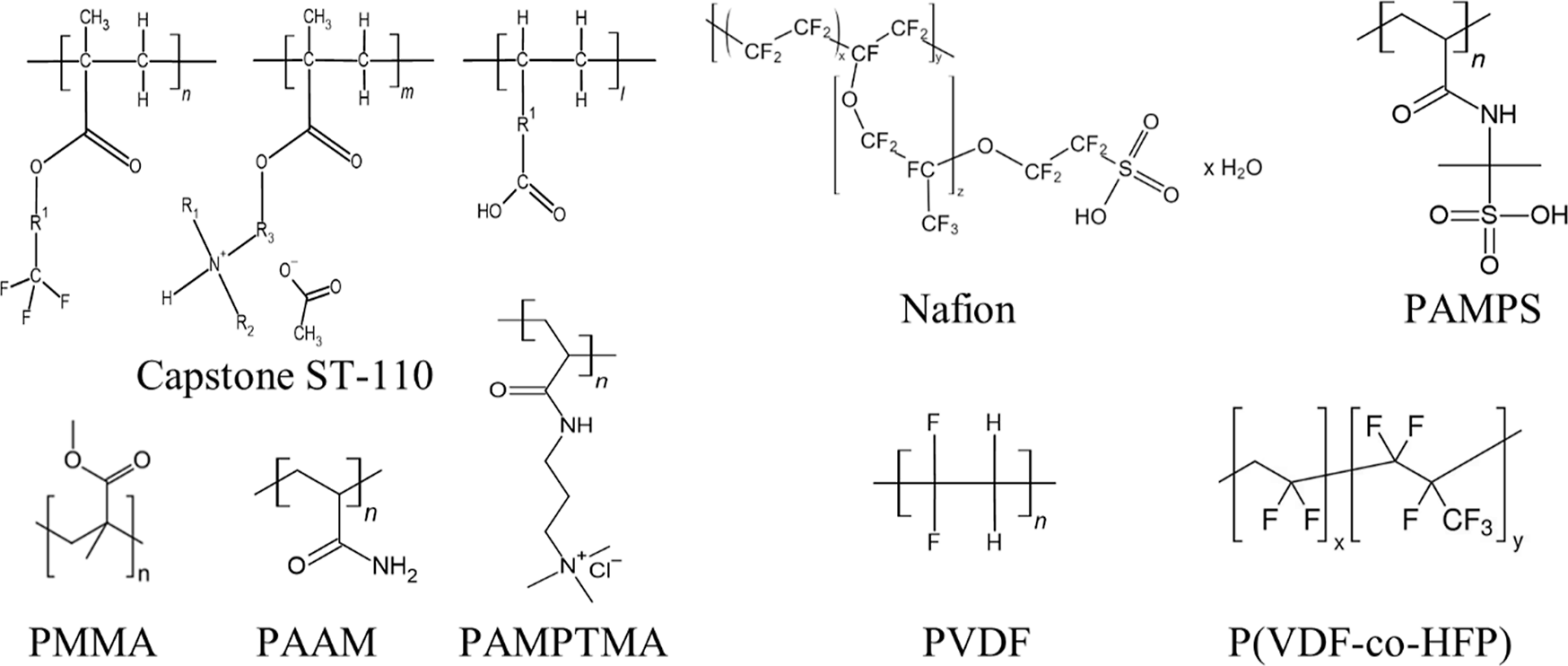
Structure of the different polymers used as cathode catalyst binders in this study.

Aqueous dispersion of Capstone ST-110 was purchased from Chemours, while 10 wt % aqueous Nafion dispersion was purchased from Fuel Cell Store. All other polymer binders (see Table 1 and Figure 1) were purchased or synthesized as solid powders and dissolved in suitable solvents. Ultrapure water (18.2 MΩ cm) was used for the experiments, freshly produced using a Millipore Direct Q3 UV instrument. A 4.7 purity CO (from Messer) cylinder was used for the CORR studies. Copper nanoparticles (nominal particle size of 80 nm, 99.9%+ purity) were purchased from Nanografi Nano Technology, while Iridium black was purchased from Fuel Cell Store. Nickel foams (applied as anode) were purchased from Recemat BV and were activated by soaking them in 1 M HCl solution for at least 10 min prior to use (after rinsing with large amounts of deionized water).

**Table 1:** Polymers Used in This Study as Cathode Catalyst Binders^a^

polymer full name	abbreviation	used solvent for dispersion	supplier
Capstone ST-110	Capstone	3:1 H_2_O/IPA	Chemours
Nafion	Nafion	3:1 H_2_O/IPA	Fuel Cell Store
poly(2-acrylamido-2-methyl-1-propanesulfonic acid)	PAMPS	EtOH	Faculty of Chemistry and Chemical Engineering University of Maribor
poly(acrylamido-propyl-trimethylammonium chloride)	PAMPTMA	EtOH	Faculty of Chemistry and Chemical Engineering University of Maribor
polyacrylamide	PAAM	H_2_O	Faculty of Chemistry and Chemical Engineering University of Maribor
poly(methyl methacrylate)	PMMA	DMSO	Sigma-Aldrich
poly(vinylidene fluoride)	PVDF	DMSO	Apollo Scientific
poly(vinylidene fluoride-*co*-hexafluoropropylene)	P(VDF-*co*-HFP)	DMSO	Sigma-Aldrich

a IPA: isopropanol, DMSO: dimethyl sulfoxide, EtOH: ethanol.

PAMPS, PAMPTMA and PAAM were prepared according to our former reports^42,43^ briefly. PAAM was prepared from a 0.25 M solution of acrylamide (AAM) (355 mg, 5.00 mmol) in MQ water (20 mL). To this solution ammonium persulphate (APS) (2 wt %; 7.1 mg, 0.031 mmol) and *N*,*N*,*N*′,*N*′-tetramethylethane-1,2-diamine (TEMED) (1 wt %; 2.80 μL, 2.17 mg, 0.019 mmol) were added as initiators to ensure polymerization. The reaction mixture was heated to 40 °C in an oil bath for 24 h. After polymerization the polymer was precipitated from THF/EtOAc solution, centrifuged, and dried in a vacuum oven.

PAMPS was prepared from a 0.25 M solution of 2-acrylamido-2-methylpropane sulfonic acid (AMPS) (1.035 g, 4.99 mmol) in MQ water (20 mL). To this solution ammonium persulphate (APS) (2 wt %; 20.7 mg, 0.091 mmol) and *N*,*N*,*N*′,*N*′-tetramethylethane-1,2-diamine (TEMED) (1 wt %; 13.4 μL, 10.4 mg, 0.089 mmol) were added as initiators to ensure polymerization. The reaction mixture was heated to 40 °C in an oil bath for 24 h. After polymerization the polymer was precipitated from THF/EtOAc solution, centrifuged, and dried in a vacuum oven.

PAMPTMA was prepared from a 0.25 M solution of (3-acrylamidopropyl)trimethylammonium chloride (AMPTMA) (1.033 g, 5.00 mmol) in MQ water (20 mL). To this solution ammonium persulphate (APS) (2 wt %; 20.7 mg, 0.091 mmol) and *N*,*N*,*N*′,*N*′-tetramethylethane-1,2-diamine (TEMED) (1 wt %; 13.4 μL, 10.4 mg, 0.089 mmol) were added as initiators to ensure polymerization. The reaction mixture was heated to 40 °C in an oil bath for 24 h. After polymerization the polymer was precipitated from THF/EtOAc solution, centrifuged, and dried in a vacuum oven.

Gas diffusion electrodes (GDEs) were formed by spray-coating, using dispersions containing copper nanoparticles (25 mg/cm^3^) and the respective polymer binder, in varied amounts (expressed in wt %, related to the total mass of the polymer + Cu nanoparticles). For all polymers, first clear solutions were formed, and the Cu nanoparticles were only added subsequently. GDEs for studies in the microfluidic electrolyzer cell were formed on Freudenberg H23C6 GDLs, while Sigracet 28BC was used for the measurements in zero-gap electrolyzer cells. Different GDLs are used in the different electrolyzer cells based on our former experience. Shortly, we found that GDLs with crack-free microporous layers are more suitable for microfluidic application, as flooding is observed at higher current densities (under identical conditions). On the other hand, GDLs with large amounts of cracks in the microporous layers are less prone to flooding in zero-gap electrolyzers. We assume that this is related to the fact that excess water in the catalyst layer can exit through these cracks, and hence will not accumulate in the catalyst layer.

GDEs were formed by spray-coating the respective dispersions on preheated GDLs. In all cases, the catalyst loading was maintained at *m*_Cu+binder_ = 1 ± 0.1 mg cm^–2^. The GDEs were cut to size before cell assembly using a medical scalpel and 3D-printed frames.

A Biologic VMP-300 instrument was used for the electrochemical measurements in microfluidic electrolyzer cells, while a TDK Lambda Genesys power supply was used for the zero-gap electrolysis experiments. In both cases, the constant flow rate of CO was maintained using a mass-flow controller (Bronkhorst). The microfluidic electrolyzer cell was used in a single-channel, membrane-less configuration, in which the electrolyte solution was fed at a constant rate (1 cm^3^ min^–1^) using a syringe pump (KF Technology NE-300) in a flow-through mode. The anolyte was supplied in the zero-gap electrolyzer cell using a peristaltic pump (ca. 70 cm^3^ min^–1^) and recirculated from a 40 cm^3^ total volume. All experiments were performed at room temperature. However, applying larger currents in the zero-gap electrolyzer cell heats up the cell to ca. 40 °C, which was not controlled during our measurements.

A Shimadzu GC-2030 Plus gas chromatograph (operated with 6.0 He carrier gas), equipped with a barrier discharge ionization (BID) detector, a ShinCarbon ST Micropacked GC Column, and an automatic 6-way valve injection system was employed. The gas flow rate was measured using an Agilent ADM flow meter. The liquid phase products were quantified using a Bruker AV-III-500-HD NMR instrument after performing a calibration for the studied compounds, and by applying DMSO and phenol as internal standards.

A Thermo Scientific Scios 2 SEM-FIB instrument was used to study the penetration of the electrolyte solution into the catalyst layers. For this study, a droplet of a 1 M KOH solution was cast on the investigated catalyst layer. After 10 min, the residual solution was gently wiped from the surface. A trench was formed using the SEM-FIB instrument. The elemental composition within the catalyst layer was investigated using a Thermo Scientific Apreo 2 instrument via EDX measurements, observing the catalyst layer (and the void in it) in a 45° angle. The wetting properties of the formed catalyst layers were characterized using an EasyDrop (Krüss) type instrument and a *V* = 10 μL droplet of 1 M KOH solution formed on the catalyst-coated side of the GDE.

## Results and Discussion

Based on our former results with the triblock-copolymer Capstone ST-110 as a potential catalyst binder for CORR,^41^ we aimed to decipher the effect of different molecular motifs. To this end, we synthesized acrylamide-based polymers bearing different pendant groups (Figure 1). To further simplify the polymer structure, we investigated the effect of incorporating linear, hydrophobic polymers into the catalyst layer, motivated by earlier results in other areas.^44,45^ The effect of incorporating Nafion and Capstone ST-110 in the catalyst layers was also investigated under identical conditions for comparison.

When comparing the effect of different polymers, an important question must first be clarified: which solvent should be used to prepare the GDEs? Originally, we intended to use exactly the same conditions for all polymers, but, due to the different solubility of the polymers in the selected solvents, we could not find a solvent (or solvent mixture) that was equally suitable for all of them. Therefore, an optimal solvent was selected separately for each polymer, in which the polymer dissolution led to clear solution formation (see Table 1). The GDEs were thus formed from dispersions of the same concentration but using different solvents. We think this to be a more fair comparison than using the same solvent for all polymers, which is optimal for some but suboptimal for others. When using the appropriate solvents, the incorporation of the respective polymer in the catalyst layer was confirmed in all cases via vibrational spectroscopy (see some examples in Figure S1).

For all polymers, we performed CORR experiments with different polymer contents in the cathode GDE. The range of 6–10 wt % polymer content was optimal for all polymers, in terms of ethylene selectivity (Figure S2). Interestingly, this does not necessarily mean a minimum in the cell voltage (Figure S3). Note that HER might proceed with lower overpotential at fully flooded electrodes (as compared to CORR), and therefore a decrease in cell voltage can indicate the nonideal composition of a GDE, as detailed below. In the following, we compare the effect of the different polymer additives at 8 wt % polymer content in the GDE.

**Figure 2:**
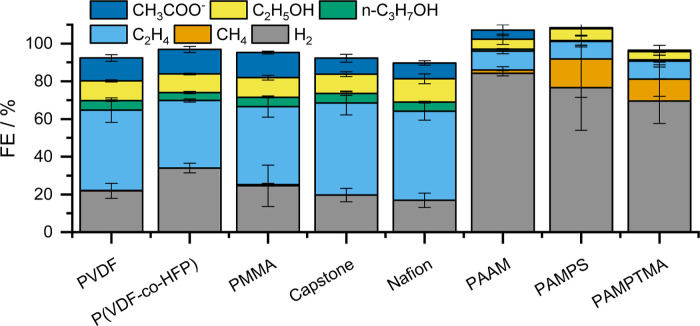
Product distribution obtained during chronopotentiometric experiments at *j* = 300 mA cm^–2^ current density, using GDEs containing Cu nanoparticles and 8 wt % of the studied polymers. The measurements were performed in a microfluidic electrolyzer cell, applying 24 cm^3^ min^–1^ CO feed at the cathode, and an electrolyte solution (1 M KOH solution) feed between the anode and the cathode at 1 cm^3^ min^–1^ rate.

The effects of the different polymers on the CORR selectivity were first investigated in chronopotentiometric measurements in a microfluidic electrolyzer cell (Figure 2). The conditions used for these measurements were chosen based on our former results,^41^ under which a high current density, selective CORR was achieved earlier using the Capstone binder.

In the case of PVDF, P(VDF-*co*-HFO) and PMMA, the determined product distributions were very close to those of the reference binders Nafion and Capstone ST-110. In these cases, the FE_H_2__ remained below 20%, while  was in the 30–35% range at *j* = 300 mA cm^–2^ total current density. Interestingly, FE_acetate_ was slightly higher with these three, as compared to the reference polymers. A notably higher FE_H_2__ was measured for the functionalized acrylamide-based polymers. Note that a quaternary ammonium group in the PAMPTMA polymer and the sulfonic acid group of the PAMPS mimic the typically used anion exchange polyelectrolytes (e.g., Sustainion) and the Nafion polymer, respectively. The low CORR selectivity indicates that these molecular motifs alone do not ensure selective CO electrolysis. In these cases, a considerable methane formation was observed. We assume that this is caused by the degradation and morphological change of the copper catalyst particles (i.e.; fragmentation or the formation of copper hydroxide).^41^

**Figure 3:**
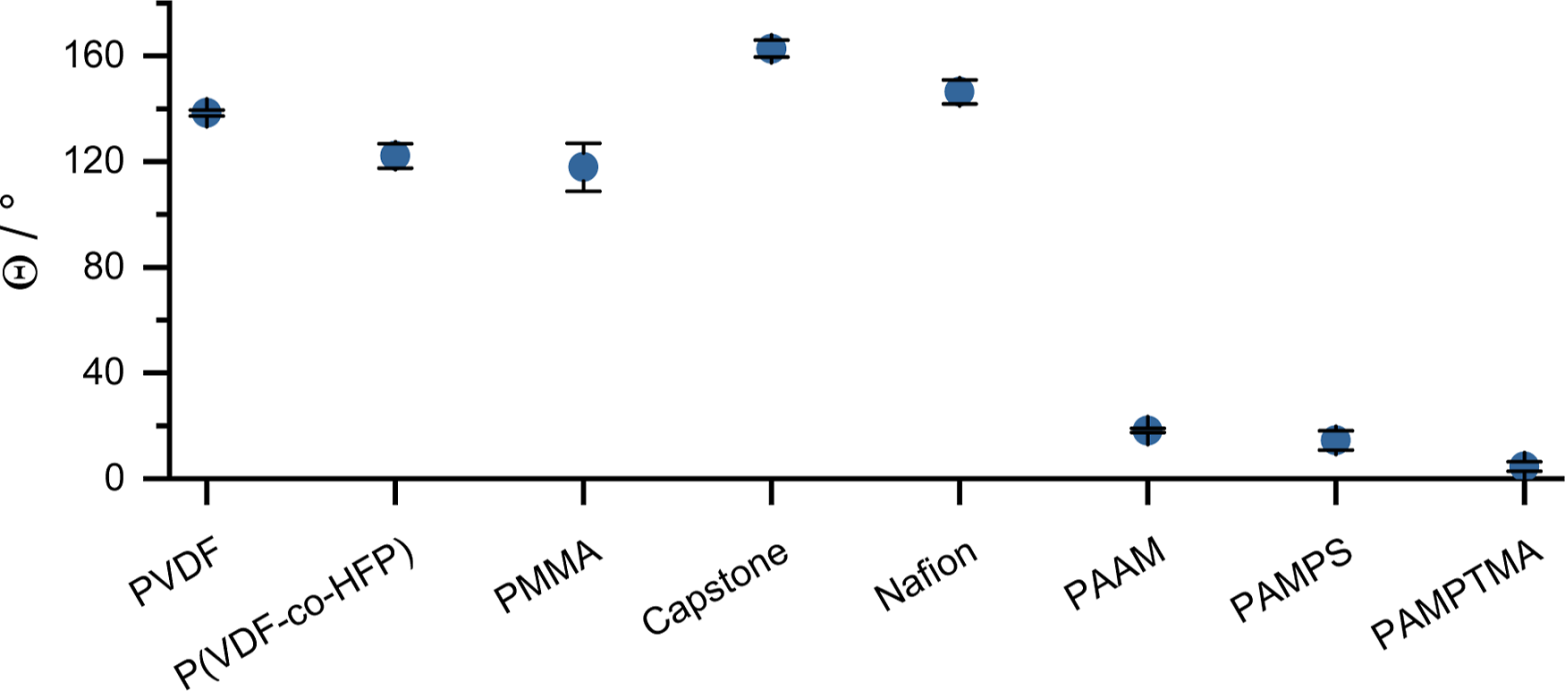
(A) Contact angles measured for the different polymer-containing catalyst layers (GDEs), applying 1 M KOH solution.

The wetting properties of the GDEs (Figure 3) corresponded well with the electrochemical results: for all polymers that ensured selective CORR, a wetting angle of at least 120° was measured (using a 1 M KOH solution). The acrylamide-based polymer containing GDEs all exhibited almost perfect wetting, and accordingly, HER occurred in these with high FE.

**Figure 4:**
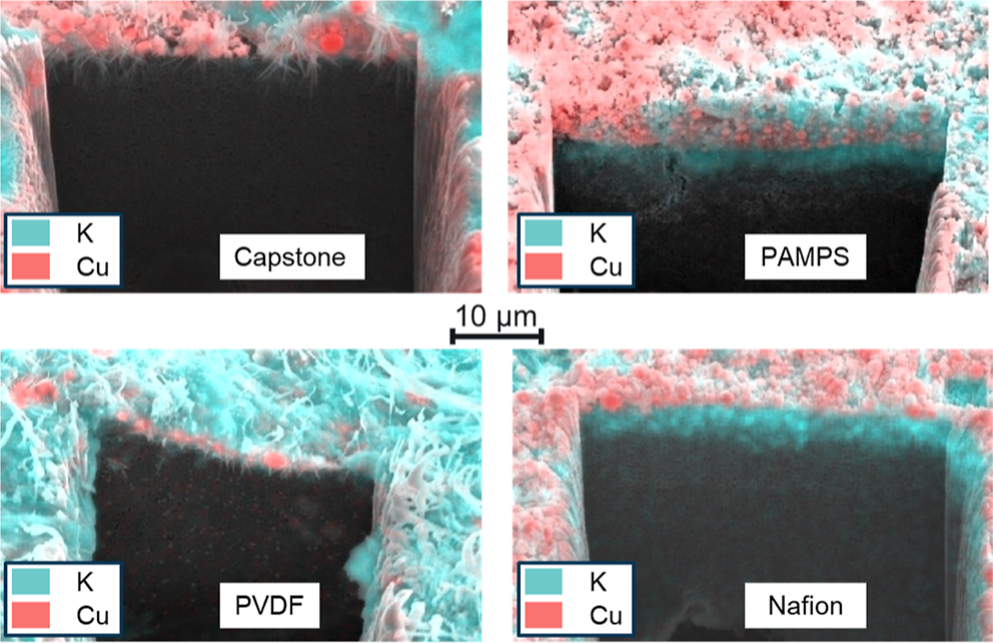
Cross-sectional SEM-EDX images of different polymers containing catalyst layers after 10 min contact time with a 1 M KOH solution.

The wetting of the catalyst layers was also examined with cross-section SEM-EDX technique (Figure 4) after a drop of 1 M KOH solution was placed on the catalyst layer and left there for 10 min; the excess (i.e., the not infiltrated part) was then removed and a trench was formed using the SEM-FIB method. Significant infiltration was observed for the polymers which led to low CORR selectivity when used as binders, while the liquid only penetrated the upper layers of the GDE with other polymers. In the Nafion-containing GDEs, large amounts of potassium ions were detected in the deeper areas of the catalyst layers, while this was proved to be a highly hydrophobic layer. This apparent contradiction can be explained by the cation-exchange properties of Nafion, which leads to the appearance of potassium ions in the deeper catalyst layer without solution penetration. All these results indicate that hydrophobicity is a key parameter in the selection of a catalyst binder for the cathode.

**Figure 5:**
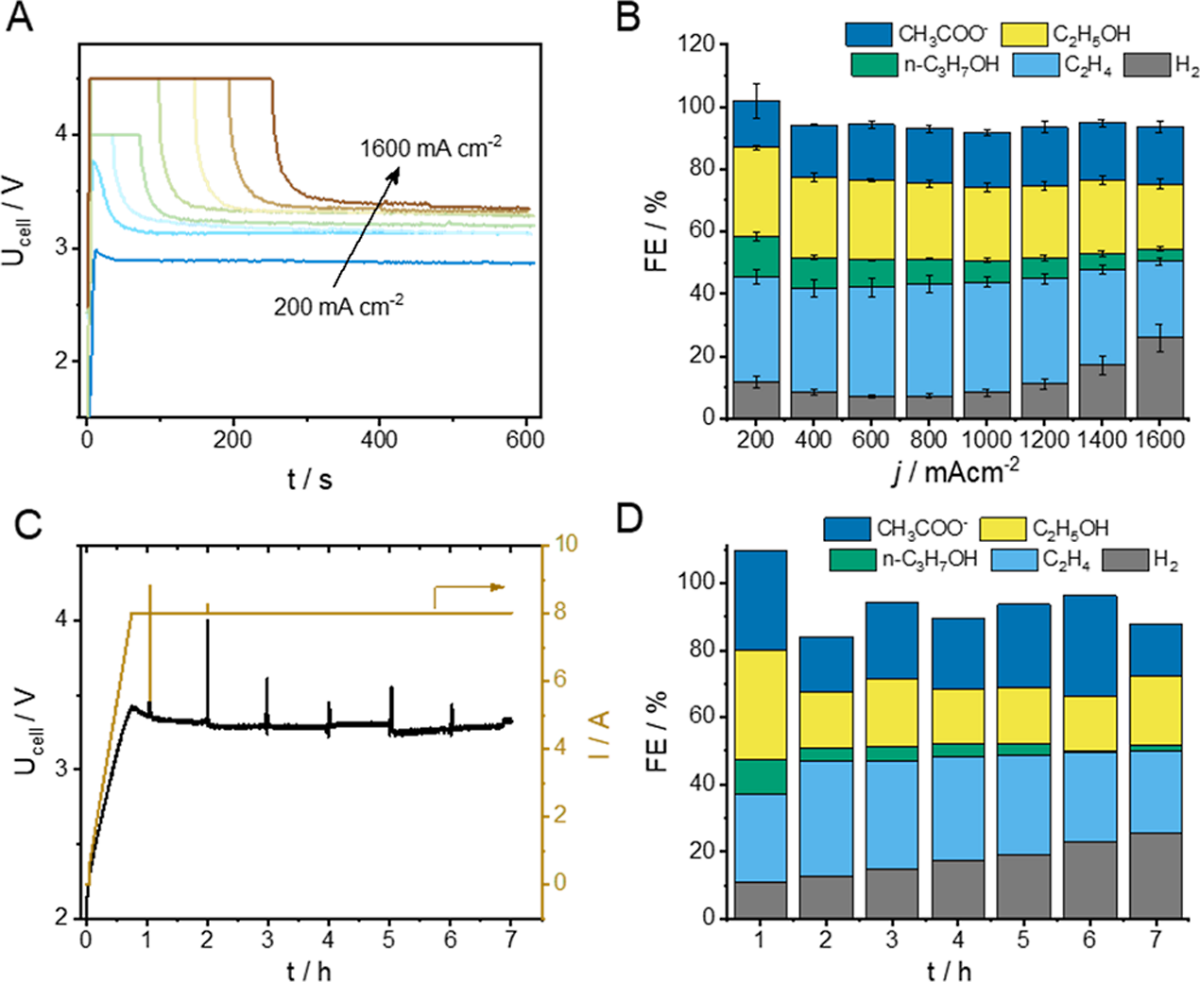
(A) Cell voltage and (B) product distribution recorded at different current densities during chronopotentiometric measurements. (C) Cell voltage and (D) product distribution during a stability test performed at *j* = 1 A cm^–2^ current density. All measurements were performed in a zero-gap electrolyzer cell, applying 100 cm^3^ min^–1^ cathodic CO feed, while 40 cm^3^ 0.5 M KOH was applied as anolyte solution, which was continuously recirculated at a rate of ca. 70 cm^3^ min^–1^. The GDE contained 8 wt % PVDF as catalyst binder.

To further demonstrate the applicability of simple linear polymers as catalyst additives in CORR, the effect of the PVDF binder was studied at higher current densities, in a zero-gap electrolyzer cell (Figure 5A,B). It should be noted that this cell architecture is better suited for studies at high current densities, and is more easily scalable, so it may represent a more realistic evaluation of each cell component for future applications.

A similar product distribution was found as in the microfluidic device but at significantly higher current densities. Importantly, the FE_H_2__ remained below 10% up to *j* = 1000 mA cm^–2^, and then gradually increased above 20% as the current density was further increased (up to 1600 mA cm^–2^). In parallel, ethylene formation occurred at ca. 35% FE up to *j* = 1000 mA cm^–2^ and only decreased beyond this current density gradually. The cell voltage, which stabilized after a few minutes of electrolysis, was only 3.35 V at the highest current density. Importantly, the PVDF containing GDEs performed very similarly to their Nafion-containing counterparts (Figure S4), in terms of both cell voltage and CORR selectivity. PMMA containing GDEs were also evaluated under identical conditions (Figure S5). In this case, higher cell voltages were recorded, but the process selectivity was similar to the other two cases at current densities up to 600 mA cm^–2^.

After a slow current ramp-up procedure (to avoid any momentary high cell voltage), the cell assembled with a PVDF-containing GDE was operated continuously at *j* = 1000 mA cm^–2^ for 6 h with periodic product sampling, and with recirculated anolyte—allowing product accumulation (Figure 5C,D). During this time, FE_H_2__ gradually increased from 10% to 25%, while the ethylene formation rate decreased at almost the same rate. Interestingly, much smaller fluctuations were observed in the selectivity for the formation of liquid products—only the selectivity of *n*-propanol formation decreased monotonously during the measurement.

## Conclusions

Simple linear polymers were tested as catalyst binders for the electrochemical reduction of carbon monoxide. We aimed to uncover the effect of different molecular motifs present in the typically used complex-structured polymers. Based on our electrochemical and electrode wetting experiments, we found a high ethylene formation selectivity for the highly hydrophobic catalyst layers. In the case of the polymers PMMA and PVDF, the selectivity for ethylene formation was very close to that measured with the polymers Nafion and Capstone ST-110 used as established references. We believe that through the functionalization of these simple linear polymers, the effect of different functional groups can be better judged, and therefore such studies are currently in progress in our laboratory.

The applicability of PVDF was further demonstrated in experiments performed in zero-gap electrolyzer cells, at high current densities, and during continuous operation for several hours. Our opinion is that instead of designing very specific molecular modifiers, the functionalization of such simple, linear polymers can be expected to enable long-term stable carbon monoxide electrolysis, and therefore we continue our experiments in this direction.

## Supporting Information Available

Containing further experimental data on the structural (FTIR) and electrochemical characterization of the formed GDEs (PDF)
