# Supplementary material for: Comparative Study of Different Polymeric Binders in Electrochemical CO Reduction
Source: Energy Fuels. 2024 Nov 5;38(22):22307–14. doi: 10.1021/acs.energyfuels.4c04058 (PMC11586900; doi:10.1021/acs.energyfuels.4c04058)
Supplement: Supplementary file 1 — ef4c04058_si_001.pdf [file ef4c04058_si_001.pdf]

– Supporting Information –

Comparative Study of Different Polymeric Binders in Electrochemical CO Reduction

Noémi V. Galbicsek,[1] Attila Kormányos,[1] Gergely Ferenc Samu[2,3] Mohd M. Ayyub,[1] Tomaž Kotnik,[4] Sebastijan Kovačič,[4,5] Csaba Janáky,[1] Balázs Endrődi[1,]*

[1]Department of Physical Chemistry and Materials Science, University of Szeged, Rerrich Square 1, Szeged H-6720, Hungary

[2]ELI-ALPS, ELI-HU Non-Profit Ltd., Wolfgang Sandner str. 3, 6728, Szeged, H-6728, Hungary

[3]Department of Molecular and Analytical Chemistry, University of Szeged, Dóm sq. 7-8, Szeged H-6721, Hungary

[4]National Institute of Chemistry, Hajdrihova 19, SI-1001 Ljubljana, Slovenia

[5]Faculty of Chemistry and Chemical Engineering, University of Maribor, Smetanova 17, SI-2000 Maribor, Slovenia

*Balázs Endrődi, Department of Physical Chemistry and Materials Science, University of Szeged, Rerrich Square 1, Szeged, H-6720 Hungary. E-mail: endrodib@chem.u-szeged.hu

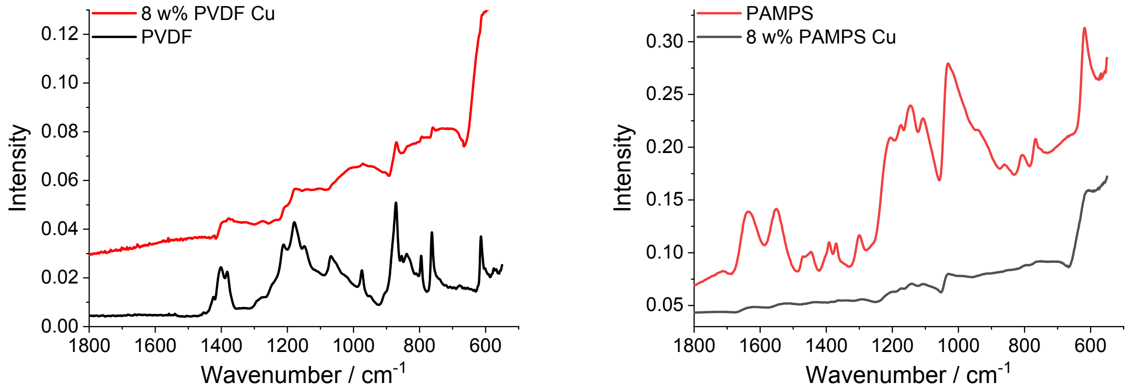

Fig. S1. Representative FTIR results proving the incorporation of the polymeric binders in the Cu catalyst layers.

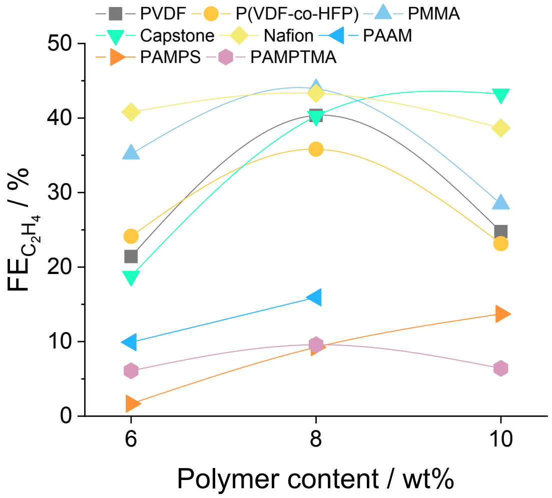

Fig. S2. Faradaic efficiency for ethylene formation with GDEs containing different polymeric binders, in varied amounts. The measurements were performed at $j = 300\ mA\ cm^{-2}$ current density in a microfluidic electrolyzer cell, applying $24\ cm^3\ min^{-1}$ CO feed at the cathode, and a

central electrolyte solution (1 M KOH solution) feed between the anode and the cathode at 1 cm^3 min^{-1} rate.

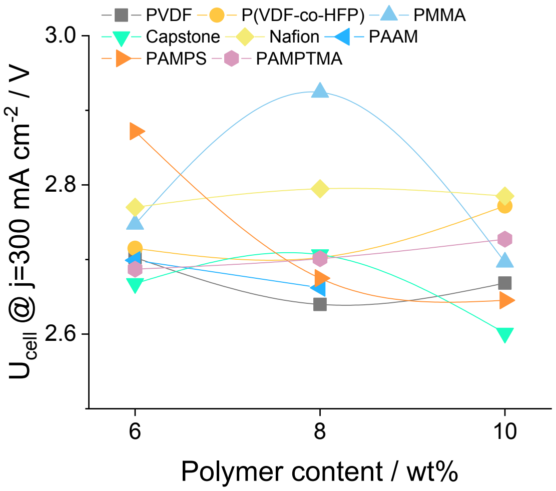

Fig. S3. Average cell voltages recorded with GDEs containing different polymeric binders, in varied amounts, at j = 300 mA cm^{-2} current density. All measurements were performed in a microfluidic electrolyzer cell, applying 24 cm^3 min^{-1} CO feed at the cathode, and a central electrolyte solution (1 M KOH solution) feed between the anode and the cathode at 1 cm^3 min^{-1} rate.

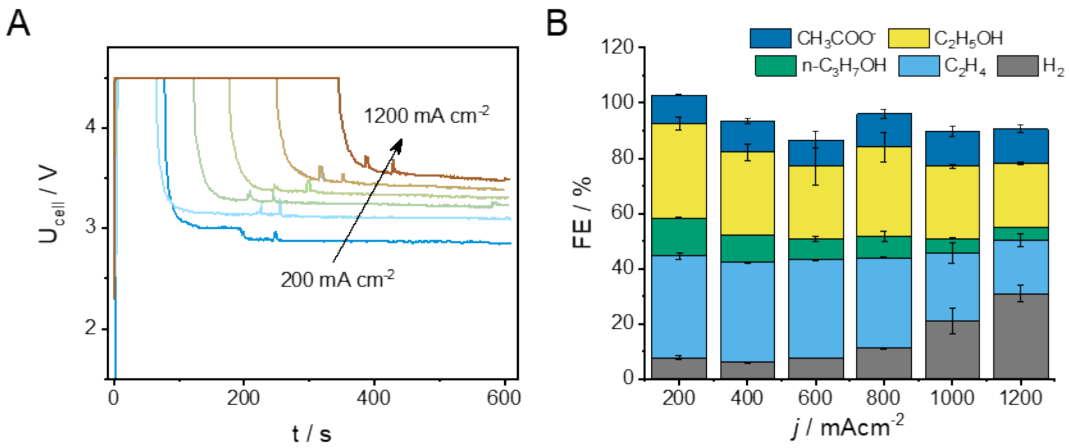

Fig. S4. (A) Cell voltage and (B) product distribution recorded at different current densities during chronopotentiometric measurements. All measurements were performed in a zero-gap electrolyzer cell, applying 100 cm³ min⁻¹ cathodic CO feed, while 40 cm³ 0.5 M KOH was applied as anolyte solution, which was continuously recirculated at a rate of ca. 60 cm³ min⁻¹. The GDE contained 8 wt% Nafion as catalyst binder.

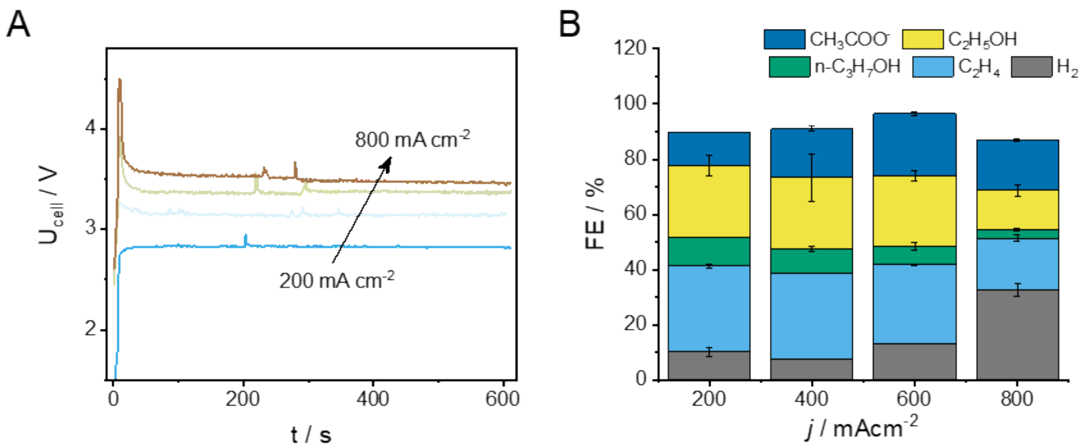

Fig. S5. (A) Cell voltage and (B) product distribution recorded at different current densities during chronopotentiometric measurements. All measurements were performed in a zero-gap electrolyzer cell, applying 100 cm³ min⁻¹ cathodic CO feed, while 40 cm³ 0.5 M KOH was

applied as anolyte solution, which was continuously recirculated at a rate of ca. 60 cm^3 min^{-1}.

The GDE contained 8 wt% PMMA as catalyst binder.
